# Cognisance of geologic hazards among Catandunganons: Promoting disaster-resilient communities

**DOI:** 10.4102/jamba.v17i1.1882

**Published:** 2025-06-30

**Authors:** Dexter M. Toyado, Patrick Alain T. Azanza

**Affiliations:** 1 College of Engineering and Architecture, Catanduanes State University, Catanduanes, Philippines; 2 Centre for Island Climate Change Solutions, Catanduanes State University, Catanduanes, Philippines; 3 Department of Administration, Catanduanes State University, Catanduanes, Philippines

**Keywords:** cognisance, awareness, disaster, resilient, community, geologic hazards

## Abstract

This study investigates the awareness of geological hazards and disaster preparedness among communities in Catanduanes, Philippines. The research employs a descriptive survey method, utilising a validated questionnaire to assess knowledge and awareness across five key areas: basic disaster knowledge, geological hazard understanding, capacity building, preparedness and response, and post-disaster management. The findings reveal a complex landscape of community awareness. While respondents show strong recognition of common disasters like earthquakes and typhoons, there is limited understanding of a broader range of geological hazards. The study identifies significant communication gaps between government agencies and the public, particularly in disseminating disaster-related information. The study also notes a concerning lack of confidence among respondents regarding their disaster preparedness and the government’s readiness to face disasters. Based on these findings, the study recommends enhancing public education and communication strategies, particularly focusing on comprehensive hazard awareness, evacuation protocols and long-term recovery processes. The research underscores the importance of leveraging modern communication platforms and addressing gender disparities in information dissemination to build a more resilient community in Catanduanes.

**Contribution:**

This study provides valuable insights for policymakers, educators and disaster management professionals, providing a foundation for targeted interventions to improve geological hazard awareness and disaster preparedness in the region.

## Introduction

Disaster, according to Qin, Feng and Zhu (2018), is a severe event that poses significant danger and results in widespread destruction, affecting both human lives and property. Disasters started to be viewed as a breakdown or flaw in development, signalling poor development and the build-up of conflicts and stresses from unsuccessful development (Wisner and Gaillard, 2009). According to the United Nations Office for Disaster Risk Reduction (UNDRR) (2017), a disaster is defined as a significant disruption to a community or society’s operations, regardless of its size. This disruption occurs when danger turns into an actual problem and interacts with existing conditions of exposure, vulnerability and limited capacity, resulting in various losses and impacts, including deaths, property, economic and environmental damages.

Disasters can be immediate, localised, widespread and long-lasting. They surpass the ability of a community to survive using its own resources, which need external assistance at local, national or international levels. The Sendai Framework for Disaster Risk Reduction (2015–2030) classifies disasters by their scale, frequency, and how quickly they begin. Small-scale disasters affect only local communities and need outside help, while large-scale disasters require national or international assistance. A disaster’s frequency depends on its likelihood and recurrence. Slow-onset disasters develop gradually, like droughts, desertification, rising sea levels and epidemics, whereas sudden-onset disasters are caused by hazardous events that appear without warning. These could be associated with geophysical, hydrological, climatological, meteorological and biological disasters. Geological or geophysical hazards, or geohazards are diverse and potentially destructive natural phenomena arising from the Earth’s geological processes. These events can inflict considerable damage on infrastructure and pose serious threats to human life (Sharif 2020).

The Philippines faces a heightened risk from these hazards because of its unique geographical and ecological characteristics (CFE-DM 2021). According to Toyado 2012, Catanduanes is prone to several geological hazards because of its geographical location within the so-called Ring of Fire as well as its topography with predominant mountainous and sloping landscape. The island experiences typhoons, earthquakes, extreme and heavy rainfall because of monsoons, and frequented by floods and landslides and tsunami warnings as a result of its exposure to the Pacific. This area is highly susceptible to natural hazards, making it a disaster hotspot. (Dilley et al. 2005). Geologically, Catanduanes is one of the earthquake-prone provinces because of its proximity to the colliding tectonic plate of the Pacific and Philippine plate. *The Philippine Disaster Risk Reduction and Management Act of 2010* consists of mitigation, preparation, prevention and response (Congress of the Philippines 2010).

This article seeks to identify and determine the level of awareness of the community in geologic hazards to create plans and activities to improve the basic understanding of geological hazards and their ability to deal with the risks associated with them.

## Research methods and design

**FIGURE 1:**
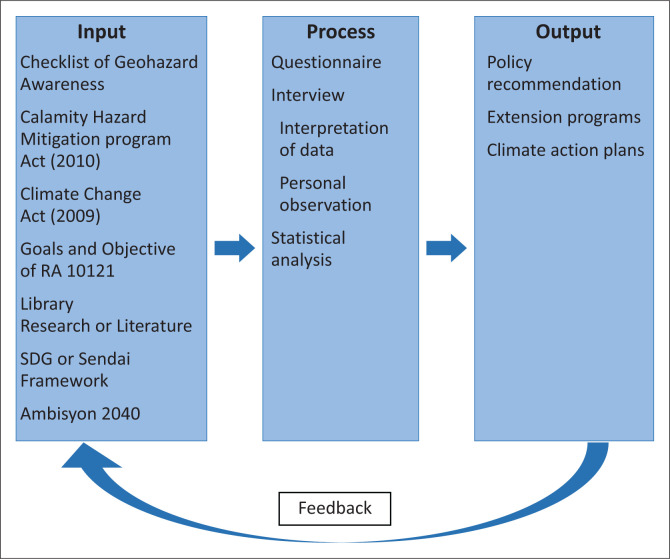
Conceptual framework of the study.

This study titled, *Cognisance of Geological Hazard Among Catanduanganons: Promoting Disaster Resilient Community*, is based on the Input-Process-Output (IPO) model. This model is a diagram that illustrates the inputs, the processing steps needed, and the resulting outputs. Figure 1 visually represents the IPO model specifically adapted for this research.

The input represents the Checklist of Geohazard Awareness, *Calamity Hazard Mitigation Program Act (2010), Climate Change Act (2009)*, Goals and Objective of RA 10121, Library Research and Literature Review, Sustainable Development Goals, Sendai Framework, and Ambisyon 2040, and other laws concerning the Philippine DRRM. For this study, the input consists of the collected data. The process involves various methods such as data gathering procedures, interviews, questionnaires, library and desktop research, data interpretation, personal observation and statistical analysis. The output will be policy recommendations, enhancement plans or action plans, and climate action plans and activities. It will also include feedback to be carried out during the process.

The research utilised the quantitative, descriptive survey method of research. According to Aggarwal and Ranganathan (2019), descriptive design is a basic type of observational study. It can either measure the spread of specific variables (quantitative descriptive research) or describe their characteristics without measurement (qualitative descriptive research). It was used to describe and analyse the existing situation about the awareness of the community with respect to the geological hazards on the island. The disaster-based questionnaire was administered among the constituents of the barangays, selected randomly from different municipalities. To get the respondents’ consent, the study’s purpose and methods were explained to them. The research used a validated, self-administered questionnaire based on existing literature on the topic. This questionnaire was designed to measure communities’ knowledge and awareness of disaster preparedness, drawing inspiration from similar studies by Mamon, Suba & Son (2017), Tuladhar et al. (2015), and Toyado (2022). The survey questionnaire utilised in this study is a validated tool for gathering data, drawing its foundation from various research studies and existing literature on geological hazard knowledge. In addition, some of its criteria were adapted from a study of Tanaka (2005) investigating the impact of disaster education on enhancing the readiness and preparedness of people in Fukui, Japan and the San Francisco Bay Area, California, USA.

This study was conducted at the barangays of the different municipalities within the province of Catanduanes, which includes the inland and coastal municipalities of the province, specifically Virac, San Andres, Pandan, Viga, San Miguel and Gigmoto. Barangays are considered as the smallest administrative division in the Philippines. The collected data from the study were organised and analysed using percentages and means. Respondents answered a total of 31 questions, which were grouped into five categories: Basic Knowledge of Disasters, Geological Hazard Related Knowledge, Geologic Hazard Capacity Building of the Community, Geologic and Disaster Preparedness and Response, and Geologic Hazard and Disaster Response, Rehabilitation, and Reconstruction. Multi-stage sampling was used for the study with the following stages: Stage 1: Randomly selecting municipalities from Catanduanes, Stage 2: Within each chosen municipality, randomly selected barangays and Stage 3: Within each selected barangay, randomly select households or individuals. A five-point Likert scale (ranging from 5 for ‘Strongly Agree’ to 1 for ‘Strongly Disagree’) was employed to gauge community responses to various geological hazard issues. The analysis of this questionnaire’s data yields a comprehensive profile of the respondents, including details about their demographics, socio-economic status and level of community involvement.

### Ethical considerations

Ethical clearance to conduct this study was obtained from the Catanduanes State University, Research Ethics Committee, dated 11 December 2023.

## Results

A total of 429 respondents were surveyed using cluster sampling, and the results reveal significant trends across various categories, including age, gender, civil status, monthly earnings, education, occupation, and roles within the barangays. Table 1 shows that respondents exhibited a high awareness, with 49% very much aware of landslides and 46.4% highly aware of earthquakes. This suggests that the community is well-aware because landslides were common in the area, because of the mountainous terrain of Catanduanes, and for earthquakes, because this is mostly discussed in school and during the national earthquake drills, which means that there is really an effective communication with these common hazards. Awareness levels were lower, with 38.7% not aware of ground settlement and 38.5% neutral or uncertain about their awareness on tsunamis. This indicates a need for more education on these hazards. There was very low awareness, with 73.9% not aware of sinkholes and 75.3% not aware of liquefaction. These hazards require significant educational efforts. A total of 43.6% were neutral or uncertain about participating in disaster awareness campaigns, suggesting low community engagement in disaster preparedness activities. A total of 34.5% were neutral awareness about their ability to predict when geologic hazards will occur, indicating uncertainty in understanding hazard forecasting. A total of 42% were neutral about knowing that disasters cannot be prevented, showing a lack of clear understanding about disaster mitigation versus prevention and only 29.6% of respondents were aware or very much aware of participating in geologic hazard mitigation and disaster risk education seminars.

**TABLE 1:** Geologic hazard-related knowledge of respondents.

Geological hazard related knowledge	Gender (percentage)									
	Strongly not aware		Not aware		Neutral		Aware		Very much aware	
	M (%)	F (%)	M (%)	F (%)	M (%)	F (%)	M (%)	F (%)	M (%)	F (%)
**I know what is geologic hazard such as:**										
Landslides	0.9	1.4	2.3	4.2	19.0	19.2	31.5	23.5	46.3	51.6
Earthquakes	0.9	0.5	3.2	4.7	19.0	21.6	31.9	25.4	44.9	47.9
Ground settlement and subsidence	0.9	0.5	44.0	33.3	32.4	35.2	8.3	14.1	14.4	16.9
Tsunami	6.5	4.2	22.7	18.3	39.4	37.6	19.9	21.1	11.6	18.8
Sinkholes	0.5	1.9	73.1	74.6	13.0	14.6	9.7	7.0	3.7	1.9
Liquefaction	2.3	2.3	75.0	75.6	17.1	17.8	4.2	3.8	1.4	0.5
I can recognize and identify geologic hazards	9.7	5.6	13.4	18.3	44.9	43.2	19.4	17.4	12.5	15.5
I participate in disaster awareness campaigns	9.3	4.7	9.7	8.0	41.7	45.5	24.5	24.4	14.8	17.4
I know when disaster from geologic hazard will happen	9.3	4.7	9.7	8.0	41.7	45.5	24.5	24.4	14.8	17.4
I know there is no prevention for the occurrence of disaster	4.6	3.3	13.9	10.3	41.7	42.3	24.1	30.0	15.7	14.1
I have been a participant in a geologic hazard mitigation and disaster risk education seminar and training	12.0	8.0	13.4	16.9	44.9	45.5	19.4	17.8	10.2	11.7

M, male; F, female.

## Discussion

A significant portion of respondents (64.8% combined) as shown in Table 2 are aware or strongly aware of the importance of sharing knowledge about geologic hazards and disasters. There is an uncertainty about government readiness to provide post-disaster assistance, with 45.9% neutral and 21% not aware. Similarly, 47.1% are neutral about the government’s technological and resource capabilities for disaster response, while 37.1% are aware or very much aware.

**TABLE 2:** Geologic hazard capacity building of the community.

Geologic hazard capacity building of the community	Gender (percentage)									
	Strongly not aware		Not aware		Neutral		Aware		Very much aware	
	M (%)	F (%)	M (%)	F (%)	M (%)	F (%)	M (%)	F (%)	M (%)	F (%)
I am aware of the significance and importance of sharing and discussing knowledge and experience of geologic hazards and disaster to everyone	3.7	0.5	2.8	3.3	30.6	29.6	35.6	34.7	27.3	31.9
I know the government is ready to provide assistance after geologic disaster	1.9	0.9	24.1	17.8	43.1	48.8	17.1	19.2	13.9	13.1
I know the government has the technology and resources to respond in geologic hazard	2.8	0.5	14.4	14.1	44.9	49.3	25.0	22.5	13.0	13.6
**I have been a participant in a seminar and training about:**										
Geologic hazards	36.6	31.5	29.6	23.5	13.4	20.2	6.0	12.7	14.4	12.2
Disaster risk reduction management	15.7	8.0	11.6	16.4	21.8	30.0	19.9	24.4	31.0	21.1
Earthquake drill	15.7	6.6	6.9	8.0	22.7	31.5	22.7	29.6	31.9	24.4
Climate change adaptation	25.5	20.2	38.0	32.4	18.1	24.9	9.7	11.7	8.8	10.8
Basic disaster preparedness and response	22.2	12.2	14.4	18.3	21.3	28.6	22.2	25.4	19.4	15.5

M, male; F, female.

This indicates a mixed perception of government preparedness. There is a concerning lack of participation, with 34% strongly unaware of attending such seminars or training. However, for specific DRRM training, there’s higher awareness (48.2% combined aware and very much aware). Earthquake drills seem to have better participation, with 54.3% combined aware and very much aware of attending such drills, but for climate change training, there is a significant lack of awareness or participation, with 58% either not aware or strongly not aware of such training.

The survey results reveal a mixed level of disaster preparedness and awareness among the respondents as shown in Table 3. A total of 72% of respondents are neutral or aware of evacuation plans, routes and shelter areas, indicating a moderate level of knowledge about evacuation procedures. There was a high awareness of early warning systems, with 69.2% of respondents being aware or very much aware. A significant majority (74.6%) are aware or very much aware of where to seek help and coordinate with agencies during and after disasters.

**TABLE 3:** Geologic and disaster preparedness and response.

Geologic and disaster preparedness and response	Gender (percentage)									
	Strongly not aware		Not aware		Neutral		Aware		Very much aware	
	M (%)	F (%)	M (%)	F (%)	M (%)	F (%)	M (%)	F (%)	M (%)	F (%)
I am aware and informed of the evacuation plans, routes, shelter areas or evacuation centers, and open space in case of a disaster	1.4	0.5	6.9	8.5	45.8	46.0	23.6	28.6	22.2	16.4
I am aware of the early warning system	1.4	0.9	4.6	2.3	25.5	26.8	35.2	41.3	33.3	28.6
I know where to ask help and coordinate with agency during and after disaster	0.9	0.5	7.9	6.6	13.9	21.1	35.2	36.2	42.1	35.7
I am ready with emergency kits in case of disaster	30.6	26.3	21.3	19.2	17.1	23.5	20.4	19.2	10.6	11.7
I am equipped with training and knowledge for disaster	30.6	26.3	21.3	19.2	17.1	23.5	20.4	19.2	10.6	11.7
I am aware about the hazards and disaster- prone areas	1.4	0.9	35.6	25.4	18.5	30.5	25.9	26.8	18.5	16.4

M, male; F, female.

This high level of awareness is crucial for effective disaster response. Responses regarding emergency kits are dispersed, with 28.4% strongly unaware and only 11.2% very much aware. This indicates a gap in personal disaster preparedness that needs addressing. Similarly, there was a lack of disaster-related training and knowledge, with 48.7% either strongly unaware or unaware. This highlights a need for more comprehensive disaster preparedness education. Knowledge of hazard- and disaster-prone areas is mixed, with 30.5% unaware and 43.8% aware or very much aware. This suggests a need for improved communication about local hazard risks.

The survey results reveal varying levels of awareness among respondents regarding government and agency actions during and after disasters. Responses about evacuation procedures are dispersed, with 27.7% aware and 27.3% not aware. There is a high level of awareness about government rescue efforts, with 69.7% of respondents either aware or very much aware. A significant portion (38.2%) is aware of the government’s provision of immediate assistance, including food, shelter and basic needs.

This indicates a general understanding of the government’s role in providing emergency relief. The majority (62.4%) are neutral about the restoration of basic services and facilities post-disaster. Similarly, 58.5% are neutral or uncertain regarding long-term reconstruction efforts as shown in Table 4.

**TABLE 4:** Geologic hazard and disaster response, rehabilitation and reconstruction.

Geologic hazard and disaster response, rehabilitation and reconstruction	Gender (percentage)									
	Strongly not aware		Not aware		Neutral		Aware		Very much aware	
	M (%)	F (%)	M (%)	F (%)	M (%)	F (%)	M (%)	F (%)	M (%)	F (%)
The government and other agencies will isolate people impacted by disaster from the impacted area after the disaster strikes (Evacuation)	0.5	0.5	30.6	23.9	20.4	24.4	24.5	31.0	24.1	20.2
The government and other agencies will provide rescue during or immediately after a disaster	0.5	0.5	3.2	1.4	25.0	30.0	43.1	37.6	28.2	30.5
The government and other agencies will provide immediate assistance, food, shelter and other basic needs to the victims	0.0	0.5	1.9	5.2	16.7	27.2	40.3	36.2	41.2	31.0
I am aware that restoration of basic services and facilities for the functioning of a community or a society affected by a disaster	0.9	0.9	5.1	5.1	17.1	56.9	42.6	22.2	34.3	14.8
I know about reconstruction and rebuilding and sustainable restoration of resilient critical infrastructures, services, housing, facilities and livelihoods to avoid or reduce future disaster risk	0.5	0.9	7.9	7.0	60.2	56.8	18.1	23.5	13.4	11.7

M, male; F, female.

The study employed inferential statistics, Pearson’s correlation analysis, to examine the relationships between geologic hazard awareness, capacity building, and preparedness and response. The analysis revealed strong positive correlations between the independent variables as shown in Table 5.

**TABLE 5:** Non-parametric correlation using Spearman’s Rho on the five different categories of the disaster questionnaire.

Correlations	RK	CBC	DPR	RRR
**Spearman’s rho RK**				
Correlation	1.000	0.660**	0.562**	0.513**
Coefficient	-	0.000	0.000	0.000
Sig. (2-tailed)	429.000	429.000	429.000	429.000
*N*	-	-	-	-
**CBC**				
Correlation	0.660**	1.000	0.567**	0.599**
Coefficient	0.000	-	0.000	0.000
Sig. (2-tailed)	429.000	429.000	429.000	429.000
*N*	-	-	-	-
**DPR**				
Correlation	0.562**	0.567**	1.000	0.721**
Coefficient	0.000	0.000	-	0.000
Sig. (2-tailed)	429.000	429.000	429.000	429.000
*N*	-	-	-	-
**RRR**				
Correlation	0.513**	0.599**	0.721**	1.000
Coefficient	0.000	0.000	0.000	-
Sig. (2-tailed)	429.000	429.000	429.000	429.000
*N*	-	-	-	-

RK, related knowledge; CBC, capacity building of the community; DPR, disaster preparedness and response; RRR, response, rehabilitation and reconstruction; sig, significance.

** , Correlation is significant at the 0.01 level (2-tailed).

The research findings as shown in Table 5 demonstrate a strong positive correlation among the variables, with correlation coefficients ranging from 0.513 to 0.721 and an average of 0.617, indicating a robust relationship. This suggests that improvements in one variable are likely to be accompanied by significant enhancements in others. Specifically, increasing knowledge about geologic hazards is strongly associated with greater preparedness and response capabilities, as well as improved rehabilitation and reconstruction efforts. Similarly, enhancing capacity building for geologic hazards is linked to corresponding increases in these areas. These relationships highlight the interconnected nature of disaster management components, emphasising that investments in education, training, and capacity building can have a cascading positive effect on overall community resilience and disaster response effectiveness. This finding underscores the importance of integrated strategies that address multiple facets of disaster preparedness and recovery simultaneously to maximise impact.

## Conclusion

The survey results revealed a mixed landscape of disaster awareness and preparedness within the community, highlighting both strengths and critical areas for improvement. While there is strong recognition of common disasters such as earthquakes, floods, and typhoons, awareness of other geological hazards such as sinkholes and liquefaction are significantly lacking. A communication gap between government agencies and the public, particularly affecting women, highlights the need for more inclusive and effective information dissemination strategies, leveraging modern platforms such as social media. Low self-perceived preparedness levels and uncertainty about government readiness further emphasise the need for enhanced education, training programmes and better communication about disaster management strategies. However, the community’s positive response to participating in disaster training presents an opportunity to boost resilience through comprehensive, inclusive and consistent capacity-building efforts that address all aspects of geologic hazards while fostering greater public confidence and engagement in disaster management.

Many people are aware of early warning systems (69.2%) and immediate rescue efforts (69.7%). However, there are gaps in knowledge about evacuation protocols (27.3% unaware), emergency kits (28.4% unaware), hazard-prone areas (30.5% unaware), and long-term recovery processes such as service restoration and reconstruction.
